# Circulating donor-derived cell-free DNA as a marker for rejection after lung transplantation

**DOI:** 10.3389/fimmu.2023.1263389

**Published:** 2023-10-11

**Authors:** Yunhui Li, Bin Liang

**Affiliations:** ^1^ Department of Laboratory Medical Center, General Hospital of Northern Theater Command, Shenyang, China; ^2^ Bioinformatics of Department, Key laboratory of Cell Biology, School of Life Sciences, China Medical University, Shenyang, China

**Keywords:** donor-derived cell-free DNA, lung transplantation, graft rejection, acute cellular rejection, antibody-mediated rejection, meta-analysis

## Abstract

**Objective:**

Recently, circulating donor-derive cell free DNA (dd-cfDNA) has gained growing attention in the field of solid organ transplantation. The aim of the study was to analyze circulating dd-cfDNA levels in graft rejection, ACR and AMR separately for each rejection type compared with non-rejection, and assessed the diagnostic potential of dd-cfDNA levels in predicting graft rejection after lung transplantation.

**Methods:**

A systematic search for relevant articles was conducted on Medline, Web of Science, China National Knowledge Infrastructure (CNKI), and Wanfang databases without restriction of languages. The search date ended on June 1, 2023. STATA software was used to analyze the difference between graft rejection, ACR, AMR and stable controls, and evaluate the diagnostic performance of circulating dd-cfDNA in detecting graft rejection.

**Results:**

The results indicated that circulating dd-cfDNA levels in graft rejection, ACR, and AMR were significantly higher than non-rejection (graft rejection: SMD=1.78, 95% CI: 1.31-2.25, *I^2 =^* 88.6%, *P*< 0.001; ACR: SMD=1.03, 95% CI: 0.47-1.59, *I^2 =^* 89.0%, *P* < 0.001; AMR: SMD= 1.78, 95% CI: 1.20-2.35, *I^2 =^* 89.8%, *P* < 0.001). Circulating dd-cfDNA levels distinguished graft rejection from non-rejection with a pooled sensitivity of 0.87 (95% CI: 0.80-0.92) and a pooled specificity of 0.82 (95% CI: 0.76-0.86). The corresponding SROC yield an AUROC of 0.90 (95% CI: 0.87-0.93).

**Conclusion:**

Circulating dd-cfDNA could be used as a non-invasive biomarker to distinguish the patients with graft rejection from normal stable controls.

**Systematic Review Registration:**

https://www.crd.york.ac.uk/prospero/, identifier CRD42023440467.

## Introduction

Lung transplantation has become the live-saving treatment for patients with end-stage pulmonary disease. Dramatic advancements have been made in surgery, medications, and postoperative management, and increased the overall survival rate to 85% and 59% at the end of the first and five years of transplantation, respectively (1). However, graft rejection, including acute cellular rejection (ACR) and antibody-mediated rejection (AMR), remains a substantial cause of morbidity and mortality after lung transplantation. ACR, as one of the most common complications, is detected in 27% of lung transplant recipients (LTRs) within the first year, and significantly related to chronic allograft dysfunction (2, 3). Moreover, due to the absence of the standardized diagnostic and treatment approaches, it is estimated that the incidence rate of AMR ranges from 4% to >50% in LTRs (4). AMR, characterized by donor-specific antibodies (DSA), innate immune infiltration, and evidence of complement activation, has been consistently identified as a contributor to morbidity, chronic lung allograft dysfunction (CLAD), and graft failure (5, 6). To date, invasive biopsy is the golden standard for diagnosis of graft rejection, performed either when rejection is suspected or for surveillance (7). However, invasive graft biopsy is difficult to practice for most LTRs in clinical settings. Apart from the possibility of sampling error, high economic cost, and physician dependence, a potential risk of complications has prevented invasive biopsy from becoming a routine screening approach (8, 9). Thus, a non-invasive, relatively objective, and sensitive biomarker is urgently needed for rejection diagnosis after transplantation.

Donor-derive cell free DNA (dd-cfDNA) refers to the free DNA released by damaged cells of graft tissue after transplantation. Recently, circulating dd-cfDNA has gained growing attention in the field of solid organ transplantation. The dd-cfDNA examination takes advantage of sensitivity and specificity of genome sequencing and the wide genomic difference between donors and recipients to quantify the circulating dd-cfDNA (10). The assay methods of dd-cfDNA includes quantitative polymerase chain reaction, digital droplet PCR, shotgun sequencing, and HLA alleles-mismatches. Xiao H, et al. summarized that dd-cfDNA can be a helpful marker for the diagnosis of AMR in transplantation recipients suspected of renal dysfunction (11). Wijtvliet VPWM, et al. showed that dd-cfDNA may be a useful marker for AMR diagnosis, but probably not for ACR (12). Mounting evidence has suggested that dd-cfDNA was a potential non-invasive marker for allograft injury and rejection and could be used for graft rejection surveillance by the International Society for Heart and Lung Transplantation (ISHLT) (13–15). To the best our knowledge, no relevant systematic review and meta-analysis evaluated the circulating dd-cfDNA levels in graft rejection, ACR, and AMR, as well as the diagnostic value in graft rejection after lung transplantation.

To date, the clinical detection of circulating dd-cfDNA levels in lung transplantation has been documented in more than 10 studies, focusing on ACR, AMR or both. However, the data on the circulating dd-cfDNA levels in graft rejection after lung transplantation are conflicting. We therefore conducted a systematic review and meta-analysis to analyze circulating dd-cfDNA levels in graft rejection, ACR and AMR separately for each rejection type compared with non-rejection, and assessed the diagnostic potential of dd-cfDNA in predicting graft rejection after lung transplantation.

## Materials and methods

This systematic review and meta-analysis was performed following the Preferred Reporting Items for Systematic Reviews and Meta-Analyses (PRISMA 2020) guidelines. Quality assessment of Diagnostic Accuracy Studies-2 (QUADAS-2) tool and Newcastle Ottawa scale (NOS) tool were used to assess the quality of included studies, respectively. The protocol was registered in the International Prospective Register of Systematic Reviews (PROSPERO; CRD 42023440467).

### Literature search

A systematic search for relevant articles was conducted on Medline, Web of Science, China National Knowledge Infrastructure (CNKI), and Wanfang databases without restriction of languages. No time restrictions were applied regarding the start date of publication, and the search ended on June 1, 2023. The search strategy was conducted using the following keywords: (“donor” OR “tissue donors”) AND (“cell-free DNA” OR “cell-free nucleic acids”) AND (“rejection”) AND (“lung transplantation” OR “lung grafting” OR “pulmonary transplant” OR “pulmonary allograft”) OR “lung transplant” OR “lung allograft”). Two reviewers (LB AND LYH) screened the titles and abstracts of articles in the initial search to identify the relevant publications. Then, full texts were obtained and reviewed in detail.

### Inclusion and exclusion criteria

Articles were considered eligible if they met all of the following inclusion and exclusion criteria. The inclusion criteria included: (1) case-control studies or cohort studies; (2) studies conducted on lung transplantation, including graft rejection or ACR or AMR cases; (3) studies assessing the circulating dd-cfDNA levels, or studies including sufficient data to calculate the number of true positive (TP), true negative (TN), false positive (FP), and false negative (FN) values. Moreover, studies that met the following criteria were excluded: (1) duplicate publications; (2) review articles, editorials, comments, and conference proceedings; (3) animal experiments; (4) essential data for pooled analysis or quality assessment were not retrievable.

Two reviewers (LB and LYH) independently selected and cross-checked the articles. Any discrepancies were resolved by discussion.

### Data extraction and quality assessment

The following data were extracted from the selected articles: first author’s name, publication year, country, study design, sample number, rejection type, control type, assay method, dd-cfDNA levels, thresholds, the area under the receiver operating characteristic curve (AUROC), and its sensitivity, specificity, TP, FP, FN, and TN.

The case‐control study or cohort study was evaluated according to the NOS tool containing eight items that could be classified into three main categories (selection, comparability, and exposure/outcome evaluation) and assigned on a score of 0-9. In the NOS assessment, scores of 7-9, 4-6, and 0-3 were considered “high quality”, “moderate quality”, and “low quality”, respectively. The quality of diagnostic studies was evaluated using QUADAS‐2 tool, which consisted of seven items covering case selection, index test, golden standard, and flow and timing. The total score ≥4 (full score= 7) indicated that the quality of the study was high.

### Statistical analysis

STATA software (version 15.0, Stata Corp) was used to analyze the original data. We performed the meta-analyses of circulating dd-cfDNA levels in patients with graft rejection, ACR, and AMR in comparison with normal stable cases. Due to different assay methods of dd-cfDNA levels, standard mean difference (SMD) along with 95% confidence interval (CI) was used to compare the difference. If the original articles only provide median and interquartile ranges (IQR), the methods suggested by Luo DH, et al. and Wan X, et al. were used to convert them into means and standard deviations (SDs) (16, 17). Given the variability in the sample characteristics, diagnostic criteria, and dd-cfDNA assay methods between studies, the chi-square *Q* test and *I* ^2^ statistics were used to determine the heterogeneity of eligible studies. If *I* ^2^ > 50% or *P* < 0.05, we considered heterogeneity to be significant and adopted a random-effects model, otherwise, a fixed-effects model was adopted. Publication bias was assessed through funnel plot asymmetry and Begg's test among the included studies. Sensitivity analysis was performed to assess the impact of single study on the overall result by serial removal of a specific study.

STATA software was used to estimate the diagnostic test accuracy of circulating dd-cfDNA in graft rejection by calculating the pooled ROC area of all studies. We performed pooled analysis of sensitivity (SEN), specificity (SPE), positive likelihood ratio (PLR), negative likelihood ratio (NLR), diagnostic odds ratio (DOR), and area under the summary receiver operating characteristic curve (AUSROC), along with the corresponding 95% CI. Deek’s funnel plot was used to assess publication bias among studies. Fagan nomogram can show the correlation between prior probability, likelihood ratio and post-test probability. The more the difference between pre-test and post-test probability, the more important the biomarker is, which can provide a reference for clinical application evaluation (18). A *P* value < 0.05 was considered statistically significant.

## Results

### Search results

**Figure 1:**
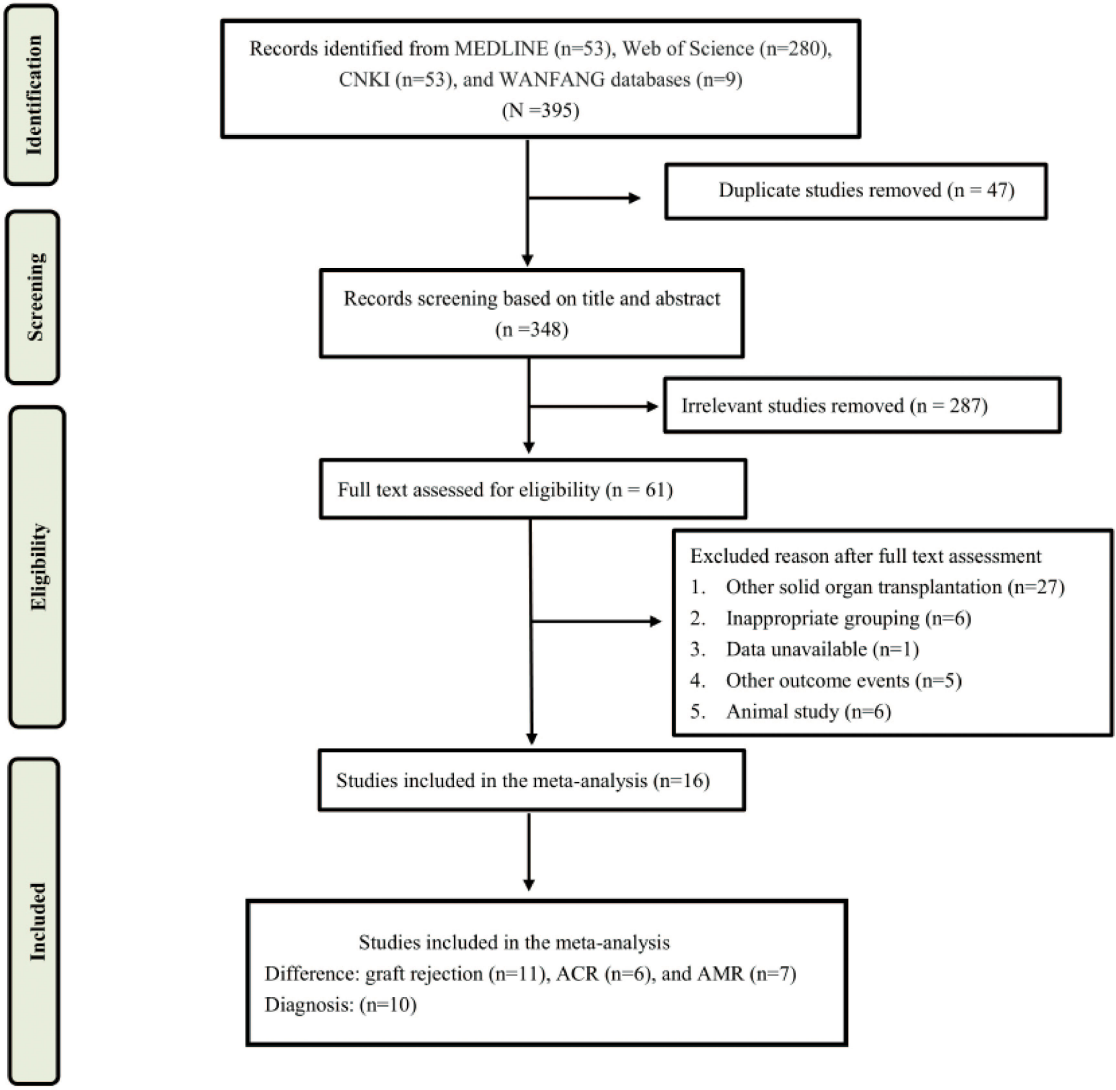
The flow chart of literature search.

The literature search and selection process were shown in **Figure 1**. A total of 395 articles were screened, including 53 from Medline, 280 from Web of Science, 53 from CNKI, and 9 from WANFANG database. After a careful review of the titles and abstracts, 304 articles were excluded for duplicate and irrelevance, of which 61 articles were selected for additional screening based on the full text. Finally, a total of 16 studies were included in this systematic review and meta-analysis (19–34).

### Study characteristics and quality assessment

**Figure 2:**
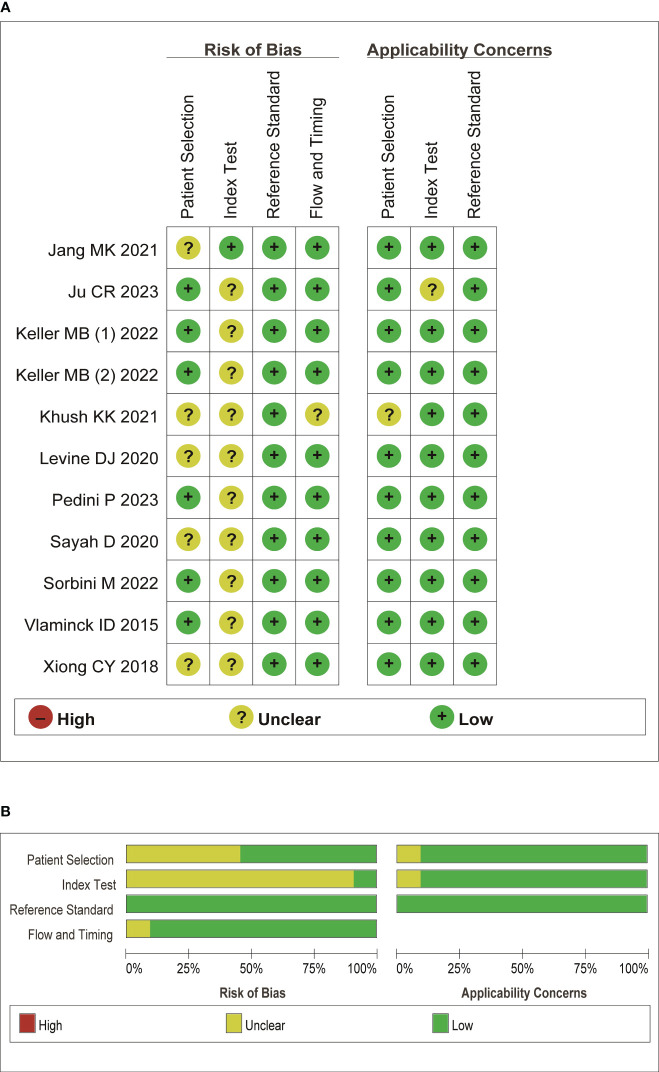
Quality assessment of the included literature. **(A)** QUADAS-2 summary plot of bias assessment. **(B)** QUADAS-2 bar plot of the individual risk of bias domains and applicability concerns.

A summary of characteristics of the included studies was shown in **Tables 1**, **Table 2**. Eleven studies were from USA (21, 23–28, 31–36), 1 study from France (20), 1 study from Italy (22), and 1 study from Japan (29), and two studies from China (19, 30). In total, eleven studies measured dd-cfDNA levels in graft rejection (19, 21, 22, 25, 26, 28–30, 32, 34), 6 studies measured dd-cfDNA levels in ACR (23–25, 27, 31, 33), and 7 studies measured dd-cfDNA levels in AMR (21, 23–25, 31, 33, 34), respectively. Eleven studies evaluated the diagnostic accuracy of circulating dd-cfDNA in graft rejection (19, 21–25, 27, 28, 30, 33), providing AUROC, sensitivity and specificity. Except for one study was retrospective study (19), all studies were prospective studies. **Table 3** and **Figure 2** present the quality results of NOS tool and QUADAS-2 tool, respectively.

**Table 1:** Characteristics of included studies in graft rejection, ACR, and AMR.

Author	Year	Country	Study design	Sample number (GR/ACR/AMR/Control)	Assay method	Reference standard
Ju CR	2023	China	retrospective cross-sectional study	GR:37 Control: 70	next-generation targeted sequencing	NA
Pedini P	2023	France	prospective study	GR:6 Control: 39	next-generation sequencing	2016 ISHLT
Keller MB	2022	USA	prospective cohort study	GR: 115 Control: 222	shotgun sequencing	2016 ISHLT
Sorbini M	2022	Italy	prospective cohort study	GR: 31 Control: 18	Expert Design assay probe panel	2016 ISHLT
Rosenheck JP	2022	USA	prospective study	ACR:27 AMR:8 Control: 99	next-generation targeted sequencing	2016 ISHLT
Khush KK	2021	USA	NA	ACR:29 AMR:9 Control: 28	targeted next-generation sequencing	2016 ISHLT
Jang MK	2021	USA	prospective cohort study	GR: 87 ACR: 30 AMR: 57 Control: 377	Shotgun sequencing	2016 ISHLT
Bazemore K	2021	USA	prospective cohort study	GR: 14 Control: 41	Shotgun sequencing	2016 ISHLT
Sayah D	2020	USA	prospective study	ACR: 13 Control: 30	next-generation sequencing	2016 ISHLT
Levine DJ	2020	USA	prospective study	GR: 21 Control: 11	next-generation targeted sequencing	NA
Tanaka S	2018	Japan	prospective study	GR: 4 Control: 6	Digital droplet PCR	NA
Xiong CY	2018	China	prospective study	GR: 13 Control: 28	Genome sequencing	NA
Agbor-Enoh S	2018	USA	prospective study	ACR: 52 AMR: 42 Control: 98	Genome sequencing	2016 ISHLT
Zhou J	2017	USA	prospective study	GR: 18 Control: 24	Droplet digital PCR	ISHLT
Sharon E	2017	USA	prospective study	GR: 38 AMR: 10 Control: 354	Genome sequencing	NA
Vlaminck ID	2015	USA	prospective study	AMR: 8 Control: 99	Genome sequencing	NA

GR, graft rejection; ACR, acute cellular rejection; AMR, antibody-mediated rejection; NA, non-available.

**Table 2:** Characteristics of included studies for diagnostic performance.

Author	Year	Country	Study design	Threshold	TP	FP	FN	TN	AUC	SEN (%)	SPE (%)
Ju CR	2023	China	retrospective cross-sectional study	1.17%	33	18	4	115	0.929(0.892-0.967)	89.19	86.47
Keller MB (1)	2022	USA	prospective cohort study	1.1%	60	11	5	54	0.89(0.82-0.97)	92	80
Keller MB (2)	2022	USA	prospective cohort study	1.1%	122	27	34	129	0.86(0.81-0.90)	78	83
Sorbini M	2022	Italy	prospective cohort study	1.25%	40	13	9	36	0.87(0.75-0.98)	80.7	73.3
Rosenheck JP	2022	USA	prospective study	1.0%	119	23	15	111	0.91(0.83-0.98)	89.06	82.86
Khush KK	2021	USA	NA	0.85%	21	9	17	29	0.667(0.586-0.738)	55.6	75.8
Jang MK	2021	USA	prospective cohort study	0.5%	441	74	23	302	0.89(0.83-0.93)	95	65
Sayah D	2020	USA	prospective study	0.87%	31	20	12	23	0.717(0.547-0.887)	73.1	52.9
Levine DJ	2020	USA	prospective study	0.51%	26	6	0	32	0.98 (0.937-1.02)	81	100
Xiong CY	2018	China	prospective study	NA	35	0	6	41	NA	84.6	100
Vlaminck ID	2015	USA	prospective study	1.0%	119	23	15	111	0.91(0.83-0.98)	89.06	82.86

TP, true positive; FP, false positive; FN, false negative; TN, true negative; AUC, area under the curve; SEN, sensitivity; SPE, specificity; NA, non-available.

**Table 3:** Quality scores of cohort studies using Newcastle-Ottawa Scale.

Study	Selection				Comparability	Outcome			NOS
	Q1	Q2	Q3	Q4	Q5	Q6	Q7	Q8	Overall score
Ju CR 2023	1	1	1	1	1	1	1	1	8
Pedini P 2023	1	1	1	1	1	1	1	1	8
Keller MB (1) 2022	1	1	1	1	1	1	1	1	8
Keller MB (2) 2022	1	1	1	1	1	1	1	1	8
Sorbini M 2022	1	1	1	1	1	1	1	1	8
Rosenheck JP 2022	1	1	1	1	1	1	1	1	8
Khush KK 2021	1	1	1	1	1	1	1	1	8
Jang MK 2021	1	1	1	1	1	1	1	1	8
Bazemore K 2021	1	1	1	1	1	1	1	1	8
Sayah D 2020	1	1	1	1	1	1	1	1	8
Levine DJ 2020	1	1	0	0	1	1	1	1	6
Tanaka S 2018	1	1	1	1	1	1	1	1	8
Xiong CY 2018	1	1	0	1	1	1	1	1	7
Agbor-Enoh S 2018	1	1	1	1	1	1	1	1	8
Zhou J 2017	1	1	1	0	1	1	1	1	7
Sharon E 2017	1	1	0	1	1	1	1	1	7
Vlaminck ID 2015	1	1	0	1	1	1	1	1	7

Q1. Representativeness of the exposed individuals; Q2. Selection of the non-exposed cohort; Q3. Ascertainment of exposure; Q4. Demonstration that outcome of interest was not present at start of study; Q5. Comparability on the basis of the design or analysis; Q6. Assessment of outcome; Q7. Adequate follow-up duration; Q8. Adequacy of follow up of cohorts.

### Circulating dd-cfDNA levels in graft rejection, ACR and AMR

**Figure 3:**
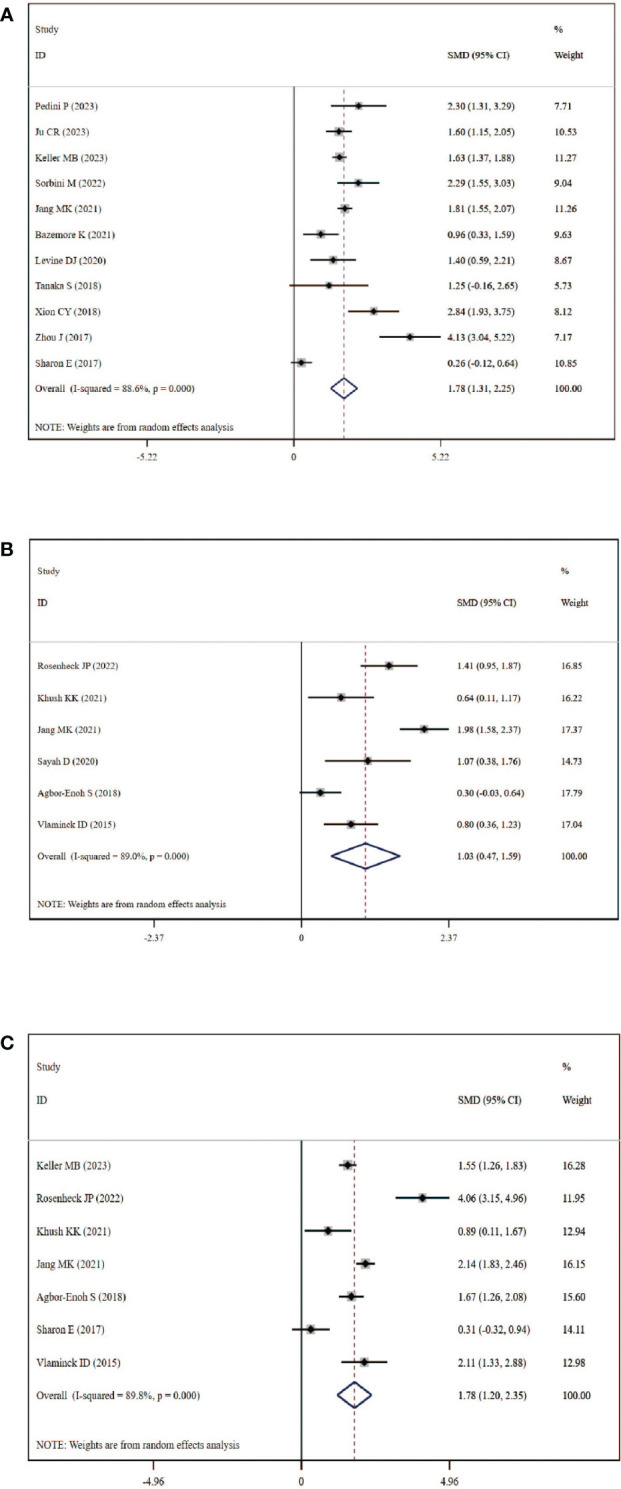
Forest plot of circulating dd-cfDNA levels in patients with rejection. **(A)** dd-cfDNA levels in graft rejection, **(B)** dd-cfDNA levels in ACR, **(C)** dd-cfDNA levels in AMR.

A total of 11 studies, including 384 graft rejection samples and 929 no-rejection samples, were included in the comparison of circulating dd-cfDNA levels in graft rejection and non-rejection groups. Due to high heterogeneity, the random-effects model was used. Meta-analysis indicted that circulating dd-cfDNA levels in graft rejection group were significantly higher than non-rejection group (SMD: 1.78, 95% CI: 1.31-2.25, *I^2 =^* 88.6%, *P*< 0.001, **Figure 3A**). Six studies including 178 samples with ACR and 731 samples without ACR showed that circulating dd-cfDNA levels in ACR group was significantly higher than non-ACR group (SMD: 1.03, 95% CI: 0.47-1.59, *I^2 =^* 89.0%, *P* < 0.001, **Figure 3B**). Moreover, seven studies including 213 samples with AMR and 1,277 samples without AMR, showed that circulating dd-cfDNA levels in AMR was significantly higher than non-AMR (SMD: 1.78, 95% CI: 1.20-2.35, *I^2 =^* 89.8%, *P* < 0.001, **Figure 3C**).

Begg’s test was used to explore the publication bias of the included studies in our meta-analysis, and a visual funnel plot was plotted to identify the publication bias (**Figure S1**). The funnel plots showed symmetrical features which indicated the absence of publication bias, identified by Begg’s tests (graft rejection: *P*=0.533; ACR: *P*=0.851; AMR: *P*=0.764). Moreover, sensitivity analysis which removed each study in turn showed that the overall result was stable and no study could influence the final result in this meta-analysis (**Figure S2**).

### Diagnostic performance of circulating dd-cfDNA in graft rejection

**Figure 4:**
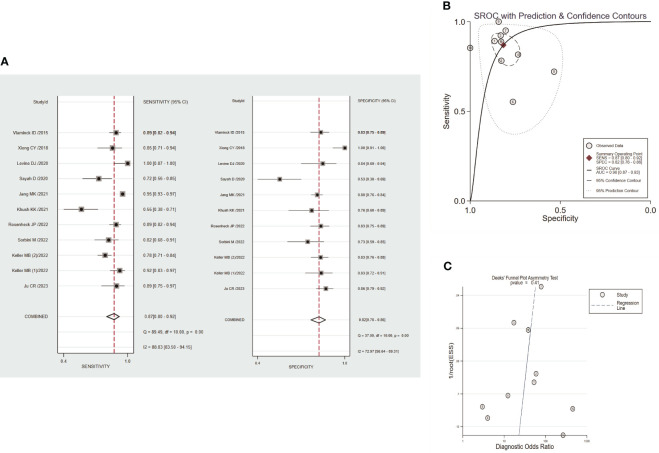
Performance of circulating dd-cfDNA in graft rejection detection. **(A)** forest plots of pooled diagnostic performance for sensitivity and specificity of circulating dd-cfDNA in graft rejection detection; **(B)** summary receiver operating characteristic (SROC) plot of circulating dd-cfDNA in graft rejection detection; **(C)** Deeks’ funnel plot for publication bias.

Circulating dd-cfDNA levels distinguished graft rejection from non-rejection with a pooled sensitivity of 0.87 (95% CI: 0.80-0.92) and a pooled specificity of 0.82 (95% CI: 0.76-0.86) (**Figure 4A**). The combined PLR, NLR, and DOR were 4.7 (95% CI: 3.4-6.5), 0.16 (95% CI:0.10-0.26), and 29 (95% CI: 14-61), respectively (**Figure S3**). The corresponding SROC yield an AUROC of 0.90 (95% CI: 0.87-0.93, **Figure 4B**), indicating high accuracy in diagnostic performance of graft rejection. There was no significant publication bias identified by Deek’s funnel plot asymmetry test (*P*=0.41, **Figure 4C**).

### Fagan nomogram

**Figure 5:**
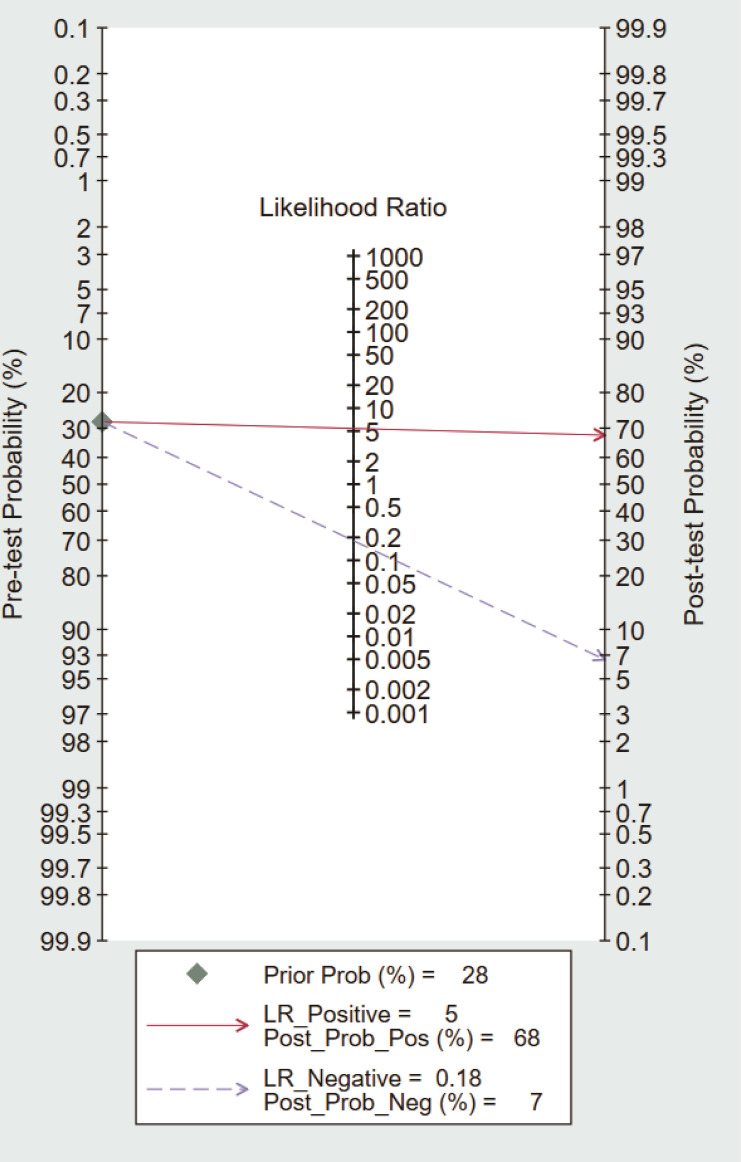
Fagan nomogram of circulating dd-cfDNA for the diagnosis of graft rejection.

To further explore the potential value of circulating dd-cfDNA level for graft rejection in lung transplantation, we built a Fagan nomogram. ISHLT reported that 28% of LTRs experience at least one episode of treated ACR within the first year (37). Assuming a 28% pre-test probability of developing graft rejection, we used circulating dd-cfDNA levels to predict a 68% post-probability of developing graft rejection and a 7% risk of not developing graft rejection (**Figure 5**). The Fagan nomogram showed that the post-probability was increased by 40% in patients with positive pre-test and decreased by 21% in patients with negative pre-test. Our findings suggest that circulating dd-cfDNA was a clinically beneficial diagnostic marker in predicting graft rejection after lung transplantation.

## Discussion

Rejection remains a significant problem following lung transplantation. In spite of advances in surgical techniques and medication, long term outcomes after lung transplantation remains worse compared with other solid organ transplantation (38). The clinical data indicated that 28% of adult recipients experienced at least one episode of acute rejection during the first year after lung transplantation (37). Currently, the biopsy with detailed pathology is regarded as the gold standard for making diagnosis based on histologic lesions associated with acute rejection. However, frequent coexistence of rejection and infection, disagreement between different pathologists, and invasive complication limited the accuracy, timeliness, and repeatability. The value of dd-cfDNA as a non-invasive biomarker for lung transplantation has been investigated in some studies. Moreover, most studies were published in the last three years, underlining the emerging interest in circulating dd-cfDNA detection as a monitor of graft rejection. However, does dd-cfDNA play a role in the current clinical paradigm for the monitoring of underlying graft rejection? In this review and meta-analysis, we found that circulating dd-cfDNA levels in lung transplantation patients with graft rejection, ACR, and AMR were higher than patients without rejection or stable patients, and circulating dd-cfDNA was a non-invasive potential marker in diagnosis of graft rejection after lung transplantatiopn.

The dd-cfDNA, detected in the blood of transplant recipients, provided huge potential as a sensitive, non-invasive, cost-effective biomarker for long-term monitoring of graft health, based on the fact that organ transplantations are also genome transplants. The plasma dd-cfDNA levels were generally low in stable transplant recipients, and dd-cfDNA levels increased due to tissue injury after solid organ transplantation (39). Snyder TM, et al. found that dd-cfDNA levels were less than 1% at stable patients, and increased to 3-4% during rejection episode after heart transplantation (40). In lung transplantation, similar results were found that dd-cfDNA had a strong concordance with clinical indicators of rejection (33). In liver transplantation, dd-cell free DNA was elevated significantly after engrafting, followed by a steady decrease to 10% of total cfDNA after 1 week (41). Moreover, dd-cfDNA accounts for <0.5% of total plasma cell free DNA in renal transplantation recipients without allograft injury, and normal dd-cfDNA could reduce the probability of detecting rejection (42). Early diagnosis of subclinical rejection might improve the clinical prognosis. Agbor-Rnoh S, et al. reported allograft injury detected by dd-cfDNA preceded clinical AMR diagnosis by a median of 2.8 months (31). The result from a total of 1,092 adult kidney transplant recipients across 7 transplant centers showed that the elevation of dd-cfDNA was significantly correlated with clinical and subclinical graft rejection (43). However, not all injurious processes may release substantial cell lysis or cause the dysregulation leading to an elevation in dd-cfDNA (44). Khush KK, et al. reported that dd-cfDNA level with AMR was no difference with stable patients after lung transplantation (24). Sayah D, et al. found that dd-cfDNA levels with ACR were not different with allograft infection (27). Moreover, dd-cfDNA values in different studies were presented as fraction (% ddcfDNA of total cfDNA) or absolute value (copies/ml plasma), which lead to varied thresholds among rejection types and clinical status. A recent study demonstrated that relative change in dd-cfDNA% has gained improved diagnostic performance compared with absolute values (45). In view of the conflicting results, we extracted the relative value of dd-cfDNA of included studies, and found that dd-cfDNA fraction levels were significantly higher in graft rejection, not only in ACR, but also in AMR in this meta-analysis.

The release of cell-free DNA into the blood is influenced by many physiological and pathological factors, such as physical and psychological stress, inflammation, and autoimmunity (46). Remarkably, dd-cfDNA was elevated in the presence of infection, in keeping with the notion that dd-cfDNA could predict graft damage. Compared with asymptomatic respiratory tract infections, symptomatic respiratory tract infections were also more likely to have high %ddcfDNA (36). A multicenter prospective cohort study of Genomic Research Alliance for Transplantation found that dd-cfDNA is a marker of pathogen associated allograft injury and may detect subclinical injury (26). In addition, very few patients underwent a prolonged decline in dd-cfDNA levels within the first 10 days after kidney transplantation, graft rejection might be suspected incorrectly due to elevated dd-cfDNA levels (47). Hence, we performed a synthesis of the currently available knowledge of circulating dd-cfDNA in relation to rejection diagnosis. The results indicated that circulating dd-cfDNA has a high accuracy in the diagnosis of graft rejection with pooled sensitivity and pooled specificity of 0.87 and 0.82, respectively. The PLR and NLR were 4.7 and 0.16, which indicated that probability of positive dd-cfDNA in graft rejection was 4.7 times higher than normal stable patients, and normal stable patients with negative results have about one-sixth chance of developing graft rejection. The DOR value was 29, indicating that circulating dd-cfDNA could effectively distinguish graft rejection from normal stable patients. Based on the Fagan’s nomogram, if the pre-test probability was 28%, the post-test probability of dd-cfDNA’s positive diagnostic result for graft rejection was 68%. In addition, the SROC curve showed that corresponding AUC was 0.90 (0.87-0.93), which was much higher than the common diagnostic criteria (>0.8). Above results indicated that circulating dd-cfDNA has a promising diagnostic performance in predicting graft rejection. However, the heterogeneity in these studies was considerable. The clinical characteristics of patients, methodological differences, cut-off values, and sample preparation protocols may lead to hidden bias in the pooled estimation. In view of no sufficient studies to perform subgroup analysis, in depth analysis of heterogeneity was not possible. With the advances in laboratory technology and knowledge, the subgroup analysis of circulating dd-cfDNA levels is needed in the future.

The present study has some limitations. First, the sample size was relatively small, and there are not many studies related to circulating dd-cfDNA levels in graft rejection in recent years. Second, the diagnostic criteria were not standardized in each study. Third, it was difficult to obtain the raw data for some included studies, which restricts us to investigate the diagnostic value of dd-cfDNA absolute quantification. Forth, owing to the different measurement methods, the circulating dd-cfDNA cut-off values used among the included studies were not completely consistent, and different cut-off values would affect the accuracy of the combined results to a certain extent. Most importantly, sources of heterogeneity in the results should still be considered carefully. Nonetheless, our study clearly showed that circulating dd-cfDNA was a promising marker in diagnosis of graft rejection, despite the fact that the meta-analysis has the limitations mentioned.

## Conclusion

Generally, our meta-analysis suggested that circulating dd-cfDNA was higher in patients with graft rejection, ACR, and AMR. Circulating dd-cfDNA could be used as a non-invasive biomarker to distinguish the patients with graft rejection from normal stable controls. Further evidence is required to explore the diagnostic accuracy of circulating dd-cfDNA in each type of graft rejection, thus paving the way for clinical application of circulating dd-cfDNA detection.

## Data availability statement

The original contributions presented in the study are included in the article/**Supplementary Material**. Further inquiries can be directed to the corresponding author.

## Author contributions

BL: Conceptualization, Data curation, Formal Analysis, Funding acquisition, Investigation, Methodology, Project administration, Resources, Writing – original draft. YL: Data curation, Formal Analysis, Investigation, Methodology, Resources, Software, Supervision, Validation, Visualization, Writing – review & editing.

## Supplementary material

Supplementary Figure 1 Funnel plot of the included studies to evaluate the publication bias. **(A)** graft rejection (n=10 studies); **(B)** ACR (n=6 studies); **(C)** AMR (n=6 studies).

Supplementary Figure 2 Sensitivity analysis of individual study. **(A)** graft rejection (n=10 studies); **(B)** ACR (n=6 studies); **(C)** AMR (n=6 studies).

Supplementary Figure 3 Forest plots of pooled diagnostic performance for PLR, NLR, and DOR.
