# Supplementary figures and images for: Circulating donor-derived cell-free DNA as a marker for rejection after lung transplantation
Source: Front Immunol. 2023 Oct 11;14:1263389. doi: 10.3389/fimmu.2023.1263389 (PMC10598712; doi:10.3389/fimmu.2023.1263389)

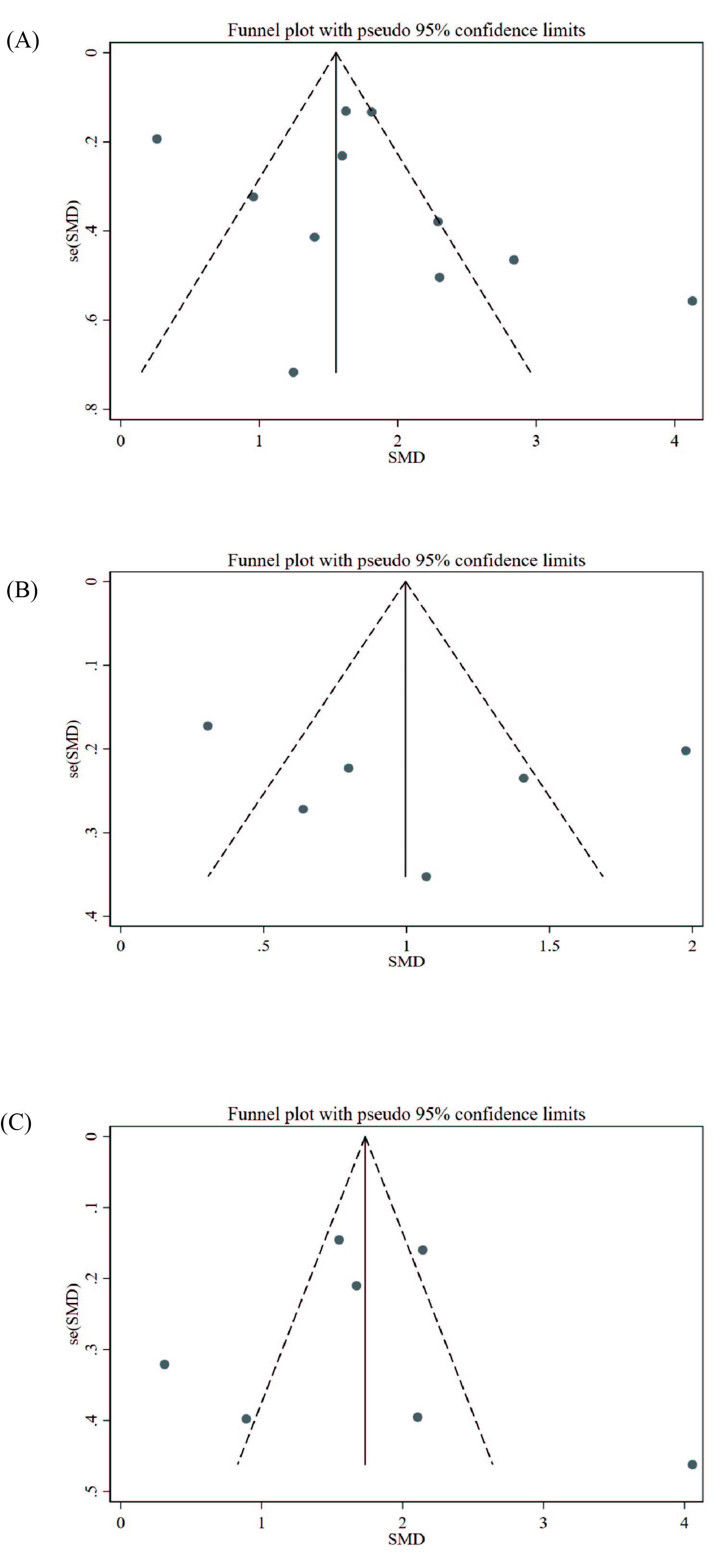

Supplement: Supplementary Figure 1 — Funnel plot of the included studies to evaluate the publication bias. (A) graft rejection (n=10 studies); (B) ACR (n=6 studies); (C) AMR (n=6 studies). [file Image_1.jpeg]

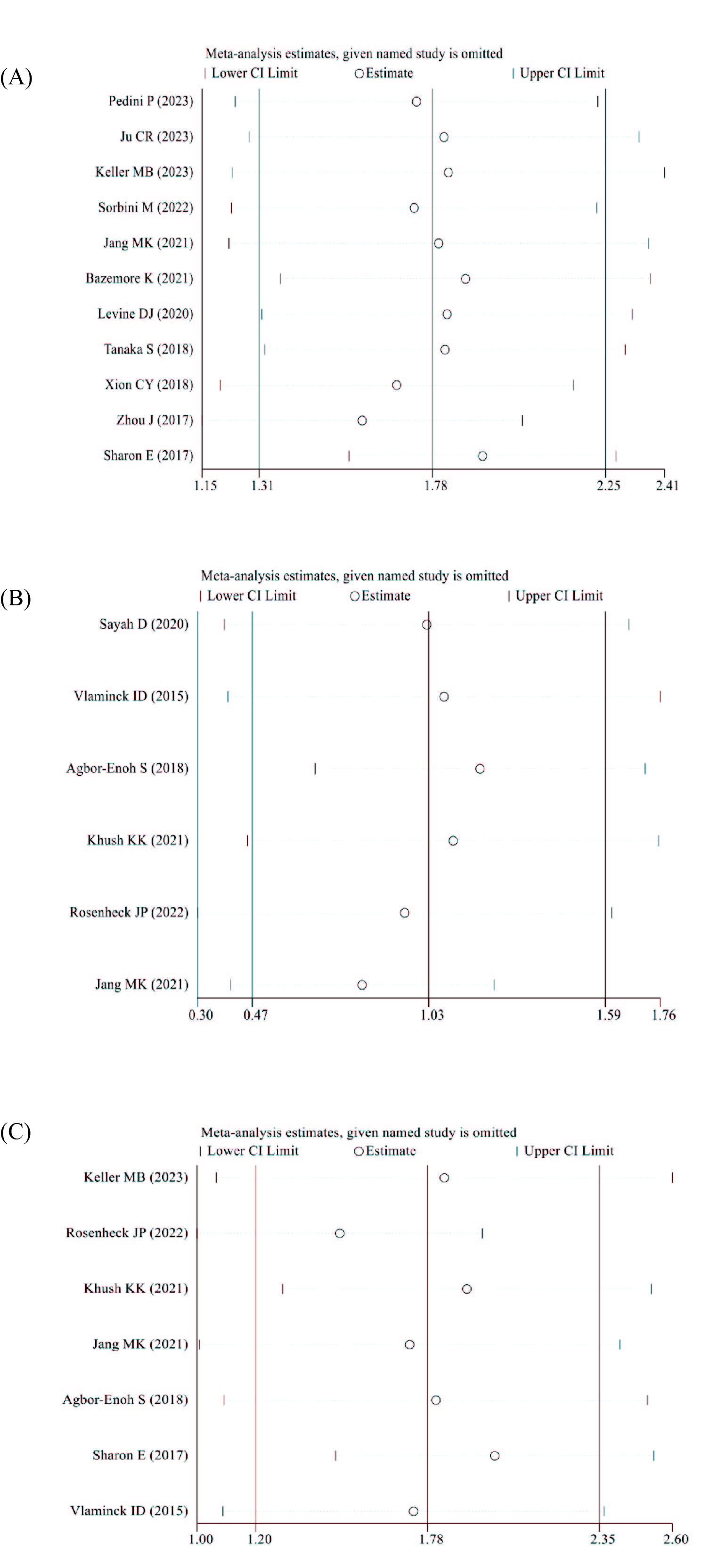

Supplement: Supplementary Figure 2 — Sensitivity analysis of individual study. (A) graft rejection (n=10 studies); (B) ACR (n=6 studies); (C) AMR (n=6 studies). [file Image_2.jpeg]

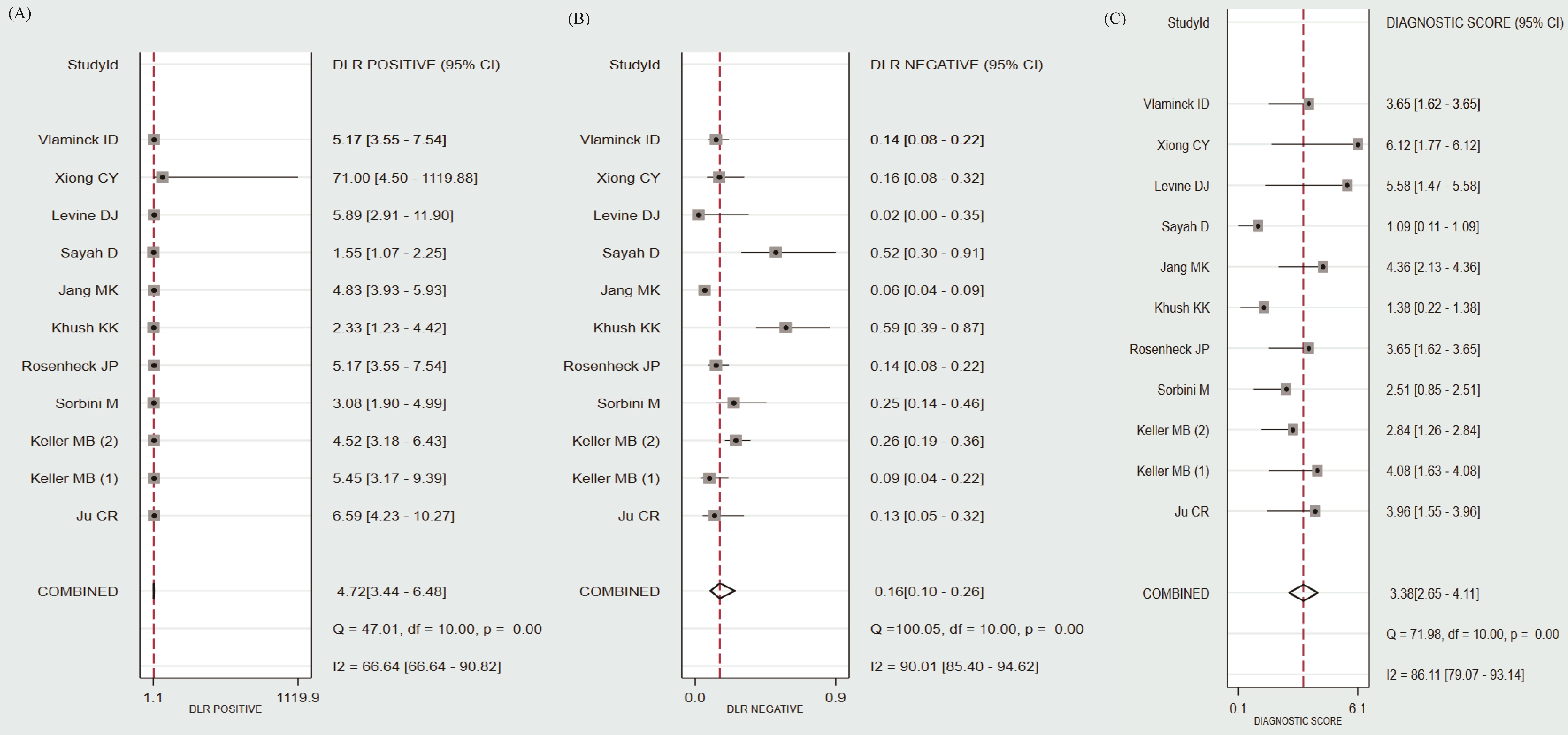

Supplement: Supplementary Figure 3 — Forest plots of pooled diagnostic performance for PLR, NLR, and DOR. [file Image_3.jpeg]
